# Liquid Chromatography-Tandem Mass Spectrometry Method for the Determination of Vardenafil and Its Application of Bioequivalence

**DOI:** 10.1155/2021/5590594

**Published:** 2021-03-25

**Authors:** Kok Zheng Gan, Riyanto Teguh Widodo, Zamri Chik, Lay Kek Teh, Mohd Salleh Rofiee, Mohd Izwan Mohamad Yusof

**Affiliations:** ^1^Department of Pharmaceutical Technology, Faculty of Pharmacy, University of Malaya, 50603 Kuala Lumpur, Malaysia; ^2^University of Malaya Bioequivalence Testing Centre (UBAT), Department of Pharmacology, Faculty of Medicine, University of Malaya, 50603 Kuala Lumpur, Malaysia; ^3^Integrative Pharmacogenomics Institute (iPROMISE), Universiti Teknologi MARA, Bandar Puncak Alam 42300, Shah Alam, Selangor, Malaysia

## Abstract

A simple, rapid, and sensitive method of liquid chromatography-tandem mass spectrometry (LC/MS/MS) method was developed and validated for the determination of vardenafil in rabbit plasma. A simple protein precipitation method with ice-cold acetonitrile was used for plasma extraction. The mass transitions m/z 489⟶151 and m/z 390⟶169 were used to measure vardenafil and tadalafil (internal standard), respectively, with a total assay run time of 6 min. The limit of detection was 0.2 ng/mL. The assay was reproducible with intra-assay and interassay precision ranging 1.17%–9.17% and 1.31%–5.86%, respectively. There was also good intra-assay and interassay accuracy between 89.3%–105.3% and 94%–102% of the expected value, respectively. The linearity range was 0.5–60 ng/mL in rabbit plasma (*r*^2^ ≥ 0.99). The measured AUC from 0 to 24 h (AUC_0 − 24*t*_) for the test and reference formulations were 174.38 ± 95.91 and 176.45 ± 76.88, respectively. For the test, *C*_max_ and *T*_max_ were 75.36 ± 59.53 ng/mL and 1.42 ± 0.19 h, whereas, for the reference, these were 58.22 ± 36.11 ng/mL and 2.04 ± 0.33 h, respectively. The test formulation achieved a slightly lower AUC_0 − 24*t*_ value (*p* > 0.05), higher *C*_max_ values (*p* > 0.05), faster *T*_max_ (*p* < 0.05), and almost equal bioavailability compared with the reference formulation.

## 1. Introduction

Vardenafil (2-[2-ethoxy-5-(4-ethylpiperazin-1-yl)sulfonylphenyl]-5-methyl-7-propyl-3H-imidazo [5, 1-f] [1, 2, 4] triazin-4-one; trihydrate; hydrochloride) is a highly potent and selective phosphodiesterase 5 (PDE5) inhibitor [1]. This is generally well-tolerated in men with erectile dysfunction (ED) with no reports of abnormal color vision at recommended doses (5–20 mg) [2]. Vardenafil was shown to have 4- to 25-fold selectivity toward PDE5 compared with sildenafil and tadalafil, which only demonstrated 10- and 5-fold selectivity, respectively [1, 2]. High-fat meals were observed to reduce vardenafil absorption if taken orally with an 18%–50% reduction in maximum concentration (*C*_max_) [3]. Vardenafil is highly bound to plasma protein (95%). The mean half-life (*t*_1/2_) of vardenafil ranges from 3.94 to 4.79 h [2]. It is extensively metabolized in the liver mainly by cytochrome P450 (CYP) 3A4 and a smaller portion by CYP3A5 and CYP2C isoforms [3]. Vardenafil film-coated tablets undergo extensive first-pass metabolism, and their absolute bioavailability is approximately 15% [4].

Formulating vardenafil in an orodispersible tablet dosage form can bypass the first-pass metabolism in the liver and can be taken even after a meal, thus improving patient satisfaction [5–7]. An integrated analysis concluded that vardenafil orodispersible tablets significantly improve erectile function in men with ED, regardless of baseline ED severity, age, or underlying conditions [8]. However, the presently marketed vardenafil orodispersible tablet takes a longer time to reach maximum concentration (*T*_max_) [5, 9]. Inappropriate use of excipients in orodispersible formulations causes the drug to mainly be absorbed via the gastrointestinal route rather than the oral transmucosal route [5]. Thus, the current orodispersible formulation should have a faster onset of action by reducing *T*_max_. Additionally, the newly optimized orodispersible formulation must also exhibit similar pharmacokinetic parameters and bioequivalence to the marketed orodispersible tablet.

In this study, there were a few challenges in quantifying vardenafil in rabbit plasma. The 10 mg dose of vardenafil ODT administered in the rabbit was low; only 150 *µ*L of plasma samples were recovered at each time point of blood collection. Most previous studies required a large sample volume to achieve a very low limit of quantification (LLOQ). Carlucci et al. (2009) developed a method to measure vardenafil in human plasma using high-performance liquid chromatography (HPLC) with ultraviolet (UV) detection [10]. However, this method was tedious and involves double extraction steps of organic solvents, also requiring a large amount of plasma (1 mL) and a high injection volume (20 *µ*L) with a running time of 15 min [10]. A validated HPLC tandem fluorescence detector was used to quantify vardenafil in rat plasma [11]. This method has a relatively long run time of 18 min and the LLOQ remained high (10 ng/mL) [11]. Abu El-Enin et al. (2015) developed a spectrofluorimetric method to quantify vardenafil in human plasma and pharmaceutical products [12]. Despite the simple protein precipitation method with acetonitrile only, this method requires a large amount of human plasma (1 mL) to achieve an LLOQ of 10 ng/mL [12].

Apart from the HPLC with UV detection, liquid chromatography with tandem mass spectrometry (LC/MS/MS) can also be used to quantify drugs and metabolites in biological samples because of its high sensitivity and selectivity. This requires only a small sample volume and has a short analytical time.

A few validated LC/MS/MS methods that quantify vardenafil in plasma have previously been published [4, 13]. A method developed by Ku et al. (2009) to measure vardenafil in human plasma requires 0.25 mL of plasma to achieve an LLOQ of 0.5 ng/mL [4]. Another method by Lake et al. (2010) requires 0.2 mL of plasma to achieve an LLOQ of 0.2 ng/mL [13]. These two methods require liquid-liquid extraction and solvent evaporation in their sample preparation [4, 13]. By contrast, Bhadoriya et al. (2018) developed a method to extract tadalafil from 0.2 mL of human plasma via solid-phase extraction [14]. The calibration curve was linear over the concentration range of 0.5–500 ng/mL [14]. Conversely, Kim et al. (2017) developed a method using acetonitrile to precipitate tadalafil from 0.02 mL of human plasma [15]. This method had achieved an LLOQ of 5 ng/mL with a total run time of 1 min [15]. The aforementioned methods require at least 200 *µ*L of plasma associated with tedious extraction methods to achieve LLOQ of 0.5 ng/mL. Thus, a new method is required to address the problem of limited samples and achieve a very low LLOQ.

## 2. Materials and Methods

### 2.1. Materials and Reagents

Vardenafil hydrochloride trihydrate (USP) was purchased from Medigene (M) Sdn Bhd, Malaysia. The purity of vardenafil hydrochloride trihydrate and tadalafil standard was all >99%. LCMS-grade acetonitrile and formic acid were also purchased from Sigma-Aldrich (M) Sdn Bhd. Standard tadalafil was obtained from the Faculty of Pharmacology, University of Malaya. Pooled blank plasma was obtained from the Animal Experimental Unit (AEU) of the University of Malaya.

### 2.2. Preparation of Standards and Quality Control (QC)

A stock solution of vardenafil (1 mg/mL) and tadalafil as Internal Standard (IS) were prepared in acetonitrile. The stock solution was further diluted with rabbit plasma to make working standard solutions in concentrations of 0.5, 1, 5, 10, 20, 30, 50, and 60 ng/mL. Internal standards with a final concentration of 40 ng/mL were prepared. The same method was used to prepare three QC samples at low (1.5 ng/mL), medium (25 ng/mL), and high (45 ng/mL).

### 2.3. Sample Preparation

A simple protein precipitation method was used to extract vardenafil and IS from plasma samples. First, 100 *µ*L of rabbit plasma was combined with 25 *µ*L tadalafil (IS) and vortex-mixed for 30 s. Then, 200 *µ*L of ice-cold acetonitrile was added to the mixture, and it was vortex-mixed again for 30 s to precipitate the protein. Subsequently, the mixture was centrifuged at 14000 rpm at 5°C for 10 min. The supernatant was collected, and 2 *µ*L of aliquot was injected directly into LC/MS/MS.

### 2.4. Chromatographic Conditions

Chromatographic separation was conducted using Agilent's Zorbax Eclipse Plus (2.1 × 50 mm i.d; 1.8 *µ*m) coupled with a guard column. Gradient elution was achieved using mobile phases consisting of 0.1% (v/v) formic acid in water (solvent A) and acetonitrile (100%, solvent B).  Table 1 describes the gradient conditions used in the method. The flow rate of liquid chromatography (LC) was maintained at 0.4 mL/min, and the total time to run the test was 6 min. The retention time for vardenafil and tadalafil was 1.56 and 1.84 min, respectively.

### 2.5. Mass Spectrometric Conditions

**Figure 1:**
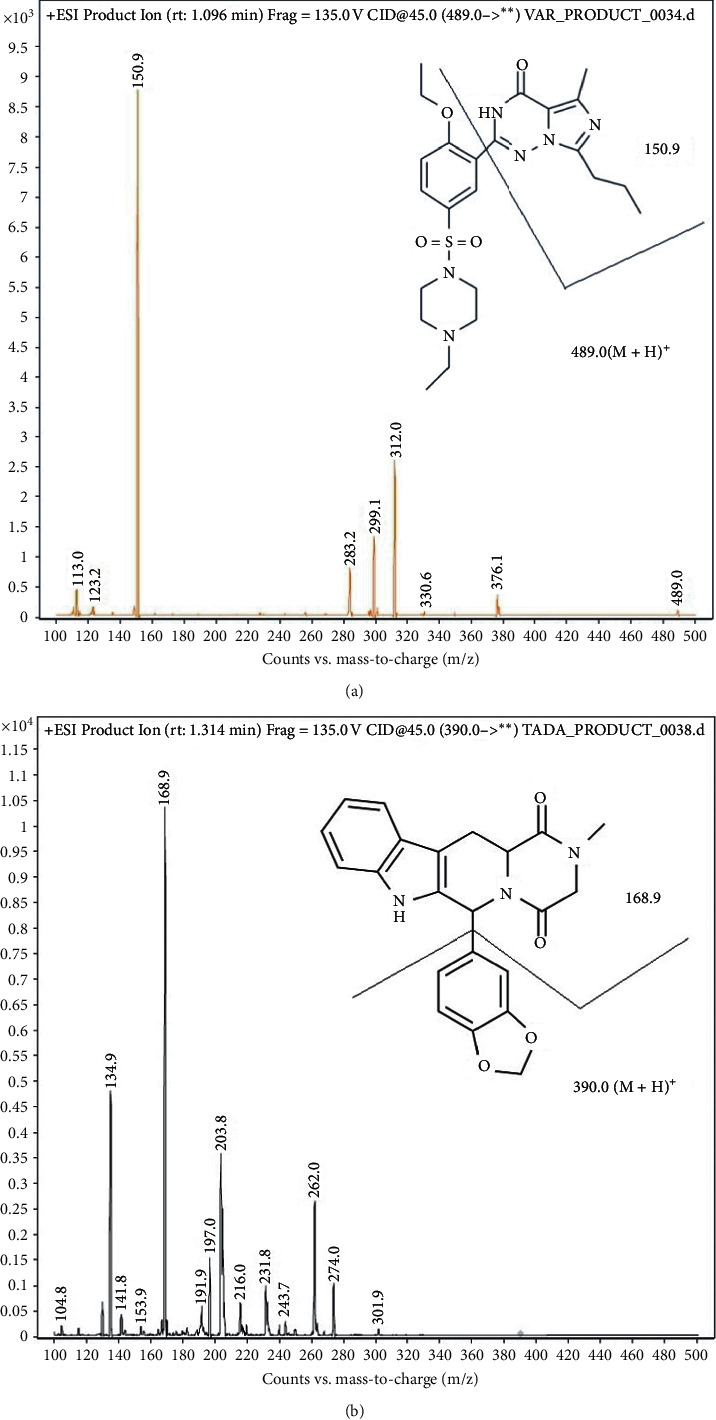
MS/MS spectrum of product ion for (a) vardenafil and (b) tadalafil in positive ion mode.

Mass spectrometry with electrospray ionization set at positive mode was used. The collision energy was set at 45 eV, cell accelerator voltage at 4 V, and the fragmentor at 135 V. The gas temperature was set at 280°C with the nebulizer at 50 psi and a 12 L/min flow rate. The aforementioned settings were determined when the highest signal was shown. The mass transitions of vardenafil were identified as m/z 489  ⟶  151 as the quantifier and m/z 489 ⟶  312 as the qualifier. Conversely, the mass transitions of tadalafil were identified as m/z 390 ⟶ 169 as the quantifier and m/z 390 ⟶ 135 as the qualifier.  Figure 1 shows the mass spectrums of both vardenafil and tadalafil.

### 2.6. Method Validation

The method was successfully validated to show the accuracy, precision, specificity, linearity, stability, and percentage of recovery from the rabbit plasma. Validation was conducted according to the Bioanalytical Method Validation: Guideline for Industry published by the United States Food and Drug Administration (USFDA) [16]. This method was validated within the concentration range of 0.5–60 ng/mL with three levels of quality control (QC) samples: QC low (1.5 ng/mL), QC medium (25 ng/mL), and QC high (45 ng/mL).

### 2.7. Animal Ethics

Animal ethics was approved by the University of Malaya Institutional Animal Care and Use Committee (UM IACUC) with Ethics Reference No: 2018-190403/PHARM/PS/GKZ. Rabbits were supplied by the AEU, Faculty of Medicine, University of Malaya.

### 2.8. Preclinical Application

**Figure 2:**
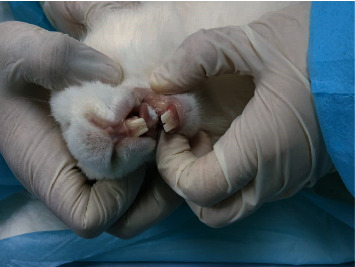
Drug administration process and fully disintegrated inside the rabbit mouth under the tongue.

The study was a randomized, single dose, balanced two-period crossover design with a 7-day washout period. Rabbits were fasted for 12 h before the experiment. Rabbits were allowed free access to water only. During the first period, six rabbits received the test formulation (newly optimized vardenafil ODT), while 6 other rabbits received the reference formulation (Staxyn® ODT 10 mg) according to a randomization schedule. After a 1-week washout period, the rabbits were switched to the other formulation accordingly. Before dosing, rabbits were sedated intramuscularly with a low dose of ketamine (10 mg/kg) and xylazine (1 mg/kg) to facilitate drug administration. Then, test or reference formulations were placed under the tongue of the rabbits, as shown in Figure 2. The rabbits were then wrapped with a towel to restrain movement before drawing 0.5 mL blood samples; these were drawn using the direct puncture technique at each time point with a 27 G × 1/2” needle from the marginal ear vein of each rabbit at 0, 0.25, 0.5, 1, 1.5, 2, 3, 4, 6, 8, 12, and 24 h after drug administration. The blood samples were immediately centrifuged at 14000 rpm at 5°C for 10 min, and plasma samples were stored at −20°C until further analysis by LC/MS/MS. At the end of the study, rabbits were euthanized and disposed of according to the laboratory protocols.

### 2.9. Bioequivalence Evaluation

ANOVA was performed on AUC_0−*∞*_, AUC0-*t*, and *C*_max_, both with and without log transformation. Bioequivalence statistics were calculated using the Pheonix WinNolin® 8.1 software to determine the 90% confidence interval range. IBM SPSS Statistics 25 software was used to determine the significance value of *T*_max_ and *t*_1/2_.

## 3. Results

### 3.1. Specificity and Selectivity

**Figure 3:**
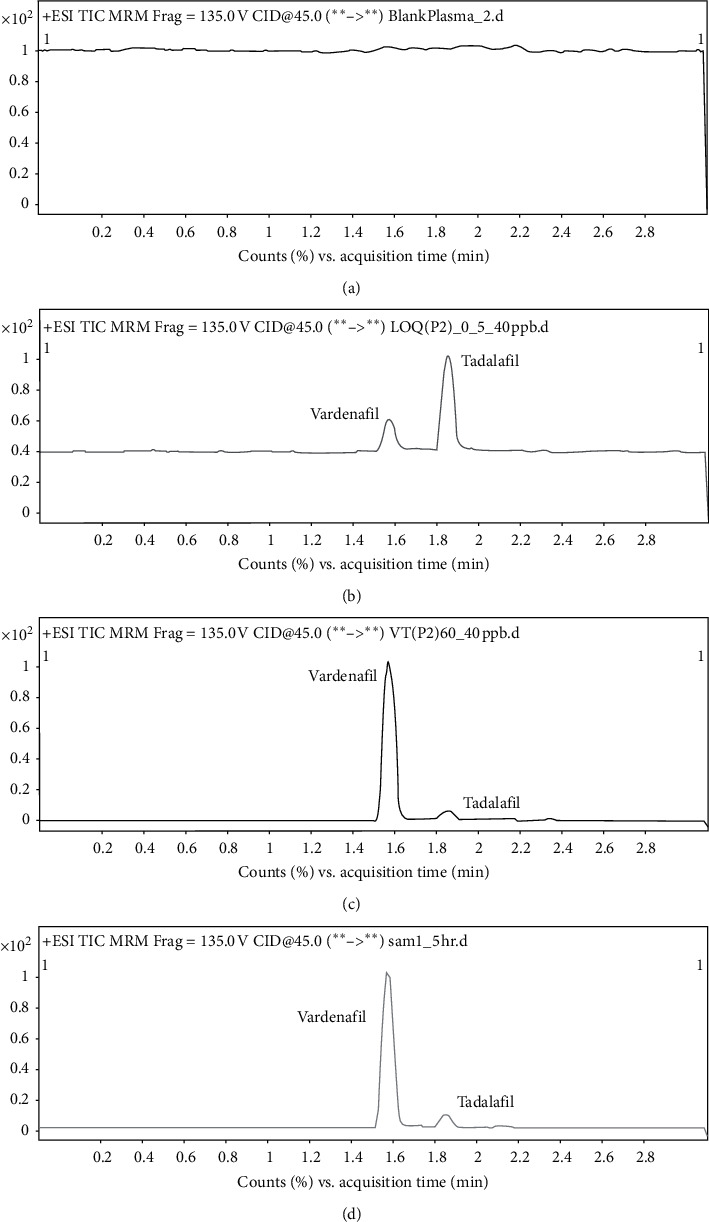
Chromatogram for (a) blank plasma with no interfering peaks at the retention time of the analytes were observed; (b) blank plasma spiked with vardenafil at LLOQ 0.5 ng/mL and IS at 40 ng/mL; (c) blank plasma spiked with vardenafil at ULOQ 60 ng/mL and IS at 40 ng/mL; and (d) plasma sample in rabbit 1.5 h after drug administration with a concentration of 44.95 ng/mL.

Six different sources (*n* = 6) of blank rabbit plasma were used to investigate the specificity and selectivity. No interfering peak was observed at the retention time of the analytes.  Figure 3(a) shows the chromatograms of blank plasma. Conversely, blank plasma spiked with LLOQ and upper limit of quantification (ULOQ) concentrations of vardenafil and IS are illustrated in Figures 3(b) and 3(c), respectively. At the retention time of vardenafil, there were no interfering peaks observed in the plasma.  Figure 3(d) shows the plasma samples 1.5 h after drug administration. The test proves that the method has high selectivity and specificity.

### 3.2. Linearity

Eight different concentrations ranging from 0.5 to 60 ng/mL were used to establish linearity via the least squares linear regression (weighted 1/×). The coefficient of determination (*R*^2^) for eight calibrators was 0.99, and the calibration curve was found to be linear. A lack-of-fit test was conducted to confirm the linearity of the method. Analysis of variance (ANOVA) test showed that the F value was less than the tabulated F value at a 95% confidence level; hence, the linear regression showed no lack of fit.

The limit of detection of vardenafil was 0.2 ng/mL, whereas the signal-to-noise ratio was 3. The lowest concentration on the calibration curve was determined as the LLOQ with the accuracy and precision achieved within ±20%. Three replicates at the lowest concentration of the calibration curve over three consecutive days were used to establish the LLOQ. The LLOQ of vardenafil was found to be 0.5 ng/mL, whereas the signal-to-noise ratio was more than 10.

### 3.3. Imprecision and Accuracy

Six replicates of LLOQ, ULOQ, and three levels of QC samples were used to determine the accuracy and precision of the method. Initially, the LLOQ, ULOQ, and three QC samples were extracted six times for one batch. Afterward, they were extracted six times in two additional batches.  Table 2 shows the results of the intraday and interday tests for imprecision and accuracy. The highest relative standard deviation (RSD) observed was 8.89%. The assay was reproducible, with an intra-assay imprecision ranging from 1.17% to 9.17% and interassay imprecision ranging from 1.31% to 5.86%. The assay exhibited good intra-assay accuracy within 89.3%–105.3% of the expected value, whereas interassay accuracy was within 94%–102% of the expected value. The results shown in Table 2 meet the acceptance criteria; hence, the imprecision and accuracy of the method are adequate.

### 3.4. Stability

Assessment of stability was conducted with three replicates of low (LQC) and high quality control (HQC) for various storage conditions. The stability of vardenafil as shown in Table 3 was tested in plasma at room temperature for 8 h, at 10°C in an autosampler for 24 h, at 20°C for three freeze-thaw cycles, and at −20°C for 2 months. To meet acceptance criteria, the RSD must be ≤15%. In this study, the highest RSD observed was 12.72%, and the mean accuracy was within 88.7%–108.4% with no significant degradation of vardenafil, as presented in Table 3.

### 3.5. Recovery

The mean recovery of vardenafil and IS from plasma was determined to be 101.4% and 70.0%, respectively (Table 4). To investigate the matrix effect, the corresponding peak areas of the postextraction spiked samples were compared with the extraction of samples from mobile phase solution at LQC, MQC, and HQC levels. The acceptance criteria for the matrix effect include an RSD of ≤15%. In this study, the highest RSD recorded in Table 5 was 4.38%. Therefore, no significant matrix effect was observed.

### 3.6. Dilution Integrity

**Figure 4:**
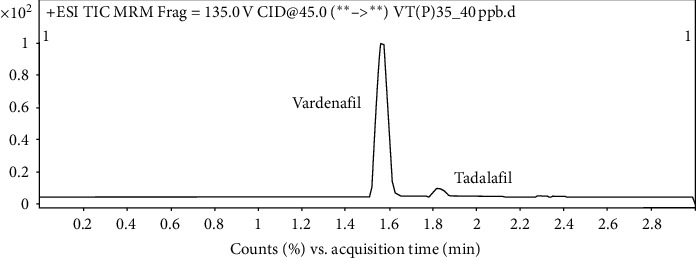
Chromatogram for plasma concentration of 35 ng/mL that diluted within the calibration range.

A dilution integrity test was conducted with six replicates of plasma samples diluted from plasma containing 350 ng/mL of vardenafil with a 1 : 10 dilution factor. The dilution was done with rabbit plasma. In this study, a concentration of 350 ng/mL was used for dilution because it covers all the detected concentration ranges, and the diluted concentration (35 ng/mL) does not overlap with any of the QCs or the calibration points. An RSD of ≤15% is needed to meet acceptance criteria. The mean accuracy and RSD were 94.85% and 8.35%, respectively (Table 6). This indicates that samples with analytes above the validated range can be diluted into the measurable ranges, as shown in Figure 4.

### 3.7. Pharmacokinetic Parameter

All pharmacokinetic parameters were calculated using noncompartmental analysis using Phoenix Win Nolin ver.8.0; these are summarized in Table 7. The value of the measured area under the curve from 0 to 24 h (AUC_0 − 24*t*_) and area under the curve from 0 to infinity (AUC_0−*∞*_) for both test and reference formulations were the same, i.e., 174.3 ± 95.91 and 176.45 ± 76.88, respectively. Conversely, the maximum concentration (*C*_max_) and time to achieve maximum concentration (*T*_max_) were 75.36 ± 59.53 ng/mL and 1.42 ± 0.19 h for the test formulation, whereas these were 58.22 ± 36.11 ng/mL and 2.04 ± 0.33 h for the reference formulation, respectively. The mean *t*_1/2_ values for the test and reference formulations were 4.83 and 4.75 h, respectively. The mean AUC_0-24*t*_ and *t*_1/2_ for the test and reference formulation were very close to each other. The mean value of *t*_1/2_ was consistent with a previous report [9]. The *C*_max_ value for the test formulation was higher than the reference formulation. The *T*_max_ value for the test formulation was lower than that for the reference formulation, indicating that the test formulation achieved the maximum therapeutic effect in a shorter amount of time.

## 4. Discussion

### 4.1. Advantages of the Method

Liquid-liquid extraction requires a drying process to enhance the resolution of the peak, which is time-consuming, requires a large amount of organic solvent, and has inconsistent recovery [17]. Solid-phase extraction is a better option with cleaner extraction compared with liquid-liquid extraction [14, 17]. However, this method requires conditioning of the column and is expensive. Comparatively, simple protein precipitation with ice-cold acetonitrile has been used in this study to extract vardenafil. This extraction method is simple, less costly, and time-saving compared with previously published methods.

This newly developed LC/MS/MS method also has high sensitivity, as it requires only a small amount (100 *µ*L) of plasma and a small injection volume (2 *µ*L) compared with the previous method. Despite the small amount of sample and injection volume, this method still can achieve a low quantification level of drug concentration (LLOQ = 0.5 ng/mL). Sample analysis can be conducted rapidly with a total running time of 6 min. Chromatographic separation was found to have better separation with gradient elution compared to isocratic elution. After 1 min of initiation, vardenafil and IS were separated with retention times of 1.56 and 1.84 min, respectively.

In this study, the intra- and interassay imprecision was RSD ≤20% for LQC and RSD ≤15% for MQC and HQC. The method exhibited good intra-assay accuracy within 89.3%–105.3% of the expected value and an interassay accuracy within 94%–102% of the expected value. Thus, this method exhibited acceptable precision and accuracy. The stability study of vardenafil shows that vardenafil was stable at room temperature for 8 h, at 10°C in an autosampler for 24 h, at 20°C for three freeze-thaw cycles, and at −20°C for 2 months.

### 4.2. Rabbit Model

The use of rabbits as models for sublingual delivery investigation has been widely established and reported in the literature. Similar to rabbits, other animals such as dogs, pigs, and monkeys are acceptable models and have nonkeratinized oral mucosa with permeability values similar to those of humans [18]. Rabbits and dogs are generally accepted as the most suitable animal models for humans because of the histological similarities in their oral cavities [19], whereas the ease of handling and animal cost makes rabbits more favorable over dogs [20]. Rabbit and human sublingual mucosa are both nonkeratinized; hence, sublingual drug delivery in rabbits can be correlated to intraoral absorption in humans [20].

### 4.3. Application of the Method

**Figure 5:**
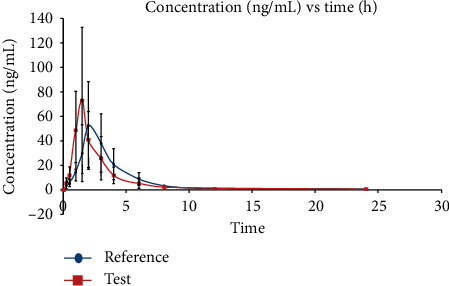
Mean plasma concentrations (ng/mL) versus time (h) of optimized formulation of vardenafil orodispersible tablet (test) and marketed orodispersible tablet formulation in New Zealand white rabbit (value expressed as mean ± SD with *n* = 12).

This newly developed and validated LC/MS/MS method was successfully applied in a pharmacokinetics study of the newly optimized formulation of vardenafil orodispersible tablet (test formulation) compared with the marketed orodispersible tablet (reference formulation) in 12 healthy rabbits.  Figure 5 plots the mean plasma concentration versus time profile for vardenafil in 12 rabbits. The test formulation was able to reach maximum concentration in the body faster than the reference formulation and has a faster onset of action. The percentages of extrapolation as shown in Figure 5 were 3.14% and 2.58% for the test and reference formulations, respectively. Low percentages of extrapolation indicate a good sampling point. Additionally, the very low LLOQ achieved in this method enabled the graph to be plotted properly.

### 4.4. ANOVA and Bioequivalence Statistics

#### 4.4.1. Area under the Curve, AUC_0-24*t*_

The 90% confidence interval (CI) for the test/reference ratio was 78.11–114.46. However, the test formulation was not bioequivalent to the reference formulation in terms of AUC because the result is not within the acceptable range of 90% CI of 80%–125% for Log 10 AUC. The acceptable range was according to the European Community's—The European Agency for the Evaluation of Medicinal Products (EC-EMEA), USFDA, and the National Pharmaceutical Regulatory Agency of Malaysia (NPRA). The Anderson Hauck probability of the result falling outside 80%–125%, which indicates the probability of a value outside 80%–125% is 0.059 (5.9 in 100). This value is larger than 0.05 (5 in 100), the level of significance, and hence, the above hypothesis cannot be rejected. Although test formulation is not bioequivalent to the reference formulation, their mean AUC_0-24t_ values were very close (174.38 and 176.45 ng·h/mL, respectively).

#### 4.4.2. Maximum Concentration, *C*_max_

The 90% CI for the test/reference ratio was 89.56–165.35. The test formulation was not bioequivalent to the reference formulation in terms of *C*_max_ because the result did not fall within the acceptable range of 90% CI of 80%–125% for Log 10 AUC. The acceptable range was according to the EC-EMEA, USFDA, and NPRA. The Anderson Hauck probability of the result falling outside 80%–125%, which indicates the probability of a value outside 80%–125%, is 0.42 (42 in 100). This value is larger than 0.05 (5 in 100), the level of significance, and hence, the above hypothesis cannot be rejected. Although the test formulation is not bioequivalent to the reference formulation, the mean *C*_max_ for the test formulation was higher than the reference formulation (75.36 and 58.22 ng/mL, respectively).

#### 4.4.3. Time to Reach Maximum Concentration, *T*_max_

The mean *T*_max_ value for vardenafil was 1.42 and 2.04 h for the test and reference formulations, respectively. Wilcoxon signed-rank test showed a statistically significant difference in terms of *T*_max_ values (*p* < 0.05) between the two. This means that the test formulation reaches its maximum concentration faster than the reference formulation. Hence, the test formulation is better than the reference formulation.

#### 4.4.4. Half-Life, *t*_1/2_

The mean *t*_1/2_ values for vardenafil were 4.83 and 4.75 h for the test and reference formulations, respectively. Paired *t*-test showed no statistically significant difference in terms of *t*_1/2_ values between the two (*p* > 0.05).

The above results prove that our newly optimized formulation achieved almost equal bioavailability to the test formulation and even achieved higher maximum concentration at a faster rate compared with the reference formulation. No significant differences were observed in *t*_1/2_ for both test and reference formulations.

## 5. Conclusion

We report on the development and validation of a simple, rapid, sensitive, and specific LC/MS/MS method for the determination of vardenafil in rabbit plasma. The major advantage of using the MS/MS system is its specificity in targeting ions of interest. This method was applied successfully to the pharmacokinetic study of vardenafil in healthy rabbits after a single dose of 10 mg vardenafil orodispersible tablet. The newly optimized vardenafil orodispersible tablet formulation exhibited similar pharmacokinetic data in terms of AUC_0-24*t*_ and *t*_1/2_. It also achieved almost equal bioavailability, higher maximum concentration, and shorter time to reach the maximum concentration compared to the reference formulation. The objective of developing a new quantification method to solve the challenges in this study was achieved.

## Figures and Tables

**Table 1:** Gradient settings of mobile phase for vardenafil hydrochloride trihydrate assay.

Time (min)	Percentage of mobile phase in eluent	
	A	B
0.0	90	10
0.5	10	90
3.0	90	10
6.0	90	10

A: 0.1% (v/v) formic acid in water; B: acetonitrile.

**Table 2:** Imprecision and accuracy for the determination of vardenafil in plasma.

	LLOQ 0.5 ng/mL	LQC 1.5 ng/mL	MQC 25 ng/mL	HQC 45 ng/mL	ULOQ 60 ng/mL
Intraday (*n* = 6)					
Day 1					
Mean	0.46	1.48	25.77	47.36	60.16
SD	0.04	2.78	9.17	7.64	4.99
RSD %	9.48	2.81	8.89	7.25	8.29
Accuracy %	92.0	98.9	103.1	105.3	100.27

Day 2					
Mean	0.50	1.50	25.14	46.52	61.73
SD	0.01	1.76	2.34	1.76	1.54
RSD %	2.00	1.76	2.33	1.71	2.57
Accuracy %	100.0	100.0	100.6	103.4	100.3

Day 3					
Mean	0.45	1.34	25.59	44.84	60.64
SD	0.03	7.06	1.17	8.65	1.77
RSD %	5.88	7.90	1.14	8.68	3.01
Accuracy %	90.0	89.30	102.4	99.6	100.1

Interday (*n* = 18)					
Mean	0.47	1.44	25.50	46.24	59.65
SD	0.03	5.86	1.31	2.86	1.93
RSD %	0.06	6.10	1.29	2.78	3.22
Accuracy %	94.0	96.1	102.0	102.8	99.41

SD: standard deviation; RSD: relative standard deviation; %: percentage; LLOQ: lower limit of quantification; LQC: low quality control; MQC: medium quality control; HQC: high quality control; ULOQ: upper limit of quantification; ng indicates nanograms; mL indicates milliliters.

**Table 3:** Stability test of vardenafil in plasma.

		LQC (AUC ratio)	HQC (AUC ratio)
Post 8 h at 25°C	Mean	0.92	29.04
	SD	0.09	1.68
	RSD %	10.68	5.79
	Accuracy %	98.2	100.1

Post 10 h at 10°C	Mean	0.85	29.92
	SD	0.04	3.25
	RSD %	4.94	10.89
	Accuracy %	90.5	103.1

Three times freeze-thaw at −20°C	Mean	0.92	31.45
	SD	0.11	0.76
	RSD %	12.72	2.44
	Accuracy %	98.1	108.4

One-month stability at −20°C	Mean	0.91	29.08
	SD	0.02	0.54
	RSD %	2.66	1.86
	Accuracy %	96.9	100.2

Two-month stability at −20°C	Mean	0.97	24.21
	SD	0.08	2.63
	RSD %	8.55	10.22
	Accuracy %	103.4	88.7

SD: standard deviation; RSD: relative standard deviation; %: percentage; LQC: low quality control; HQC: high quality control; *n* = 3.

**Table 4:** Recovery for vardenafil (standard) and internal standard.

	Standard	Internal standard
Recovery %		
LQC	99.81	74.06
MQC	103.84	67.09
HQC	100.39	68.57
Mean	101.4	70.0
SD	2.17	3.67
RSD %	2.14	5.25

SD: standard deviation; RSD: relative standard deviation; %: percentage; LQC: low quality control; MQC: medium quality control; HQC: high quality control; *n* = 3.

**Table 5:** Matrix effect of vardenafil in plasma.

	LQC (AUC ratio)	HQC (AUC ratio)
Mean	1.22	1.30
SD	0.04	1.46
RSD %	3.59	4.38

SD: standard deviation; RSD: relative standard deviation; %: percentage; LQC: low quality control; MQC: medium quality control; *n* = 6.

**Table 6:** Dilution integrity test of vardenafil in plasma.

Expected concentration	35 ng/mL
Mean %	94.85
SD	7.93
RSD %	8.35

SD: standard deviation; RSD: relative standard deviation; %: percentage; *n* = 6.

**Table 7:** Pharmacokinetic parameters of the optimized formulation of vardenafil orodispersible tablet (test) and marketed orodispersible tablet formulation (reference) in New Zealand white rabbit.

	*T*_max_ (h)	*C*_max_ (ng/mL)	AUC_0-24*t*_ (ng·h/mL)	AUC_0-*∞*_ (ng·h/mL)	*t*_1/2_ (h)
Formulation *T*					
*N*			12		
Mean	1.42 ± 0.19	75.36 ± 59.53	174.38 ± 95.91	174.38 ± 95.91	4.83 ± 1.63
Min	1.00	21.74	63.22	63.22	1.43
Median	1.50	72.79	163.12	163.12	5.12
Max	1.50	240.20	393.13	393.13	6.64
CV%	13.74	78.99	54.99	54.99	33.85
Geometric mean	1.40	59.18	150.88	150.88	4.45

Formulation *R*					
*N*			12		
Mean	2.04 ± 0.33	58.21 ± 36.11	176.45 ± 76.88	176.45 ± 76.88	4.75 ± 0.85
Min	1.50	13.15	66.47	66.47	3.42
Median	2.00	46.24	171.18	171.18	4.63
Max	3.00	140.96	295.46	295.46	5.88
CV%	16.37	62.03	43.57	43.57	17.99
Geometric mean	2.02	48.63	159.56	159.56	4.68

*R* indicates reference; *T* indicates test; *h* indicates time in hours; ng indicates nanogram; mL indicates milliliters; AUC_0-t_ indicates the measured AUC from 0 to 24 h; AUC_0-*∞*_ indicates area under the curve until infinite; *N* indicates numbers of rabbits; SD indicates standard deviation; CV indicates coefficient variation; *T*_max_ indicates time to reach the maximum peak; *C*_max_ indicates the maximum concentration that achieved; *t*_1/2_ indicates the half-life of the drug.

## Data Availability

The data used to support the finding of this study are included within the article.
